# Offending Trajectories of Men With Adult-Onset Sexual Offending Histories

**DOI:** 10.1177/10790632261429126

**Published:** 2026-02-28

**Authors:** Brandon A. Burgess, Skye Stephens, Evan McCuish, Michael C. Seto

**Affiliations:** 1 Department of Psychology, 8664 University of Manitoba, Winnipeg, MB, Canada; 2 Department of Psychology, 3690 Saint Mary’s University, Halifax, NS, Canada; 3 School of Criminology, 1763 Simon Fraser University, Burnaby, BC, Canada; 4 University of Ottawa’s Institute of Mental Health Research, Ottawa, ON, Canada

**Keywords:** sexual offending, criminal trajectories, adult-onset, group-based modeling, sexual interest in children

## Abstract

The offending trajectories of those who begin sexually offending in adulthood are poorly understood. The present study examines offending trajectories between the ages of 18 and 60 of 520 adult-onset men who were assessed at a sexual behavior clinic between 1995 and 2006. Using group-based trajectory modeling, a four-group trajectory model was retained to account for heterogeneity in the sample. The trajectories were compared on criminal career parameters (e.g., individual court contacts), victim number, and indicators of sexual interest in children (e.g., phallometric results). A trajectory with an escalating pattern of offending which onset in early adulthood was found to be associated with all three indicators of sexual interest in children and a high frequency of sexual offending. The findings of this study underscore the heterogeneity of adult-onset sexual offending, reinforcing the improbability that a one-size-fits-all approach for those who sexually offend against children would be effective.

## Introduction

Studying individual developmental periods (e.g., adolescence) ignores intra-individual change or stability between developmental periods, including how experience shapes the future. Within criminology, the criminal career paradigm provides a framework for examining patterns of change or continuity in offending across the entire life course (Cullen, 2011). Developmental and life course criminology offers perspectives on how or why long-term individual changes in traits (e.g., temperament, resilience), and external changes (e.g., employment, relationships) influence motivation or likelihood to offend over time (McGee & Farrington, 2019; Sampson & Laub, 1993; Thornberry, 2010). Patterns of change or stability in offending can be represented by a person’s offending trajectory, which captures the onset, frequency, persistence, and desistance of offending across time by measuring the offending rate across each year of age.

The study of criminal trajectories allows for a more nuanced understanding of offending patterns beyond that afforded by dichotomous indicators of recidivism. For example, the identification of criminal trajectories can help researchers view specific types of offending, such as sexual offending, within the larger context of the criminal career and its association with other types of offending (e.g., non-sexual offending). By taking a broader perspective, researchers can examine whether specific trajectories are likely to be characterized by engagement in one form of offending (specialization) or are more likely to be characterized by different types of offending (generalization). For example, a pattern of sexual offending that arises in the absence of a previous pattern of non-sexual offending may be indicative of a higher risk for sexual offending; as opposed to sexual offending preceded by a history of non-sexual offending, which may reflect a broader pattern of antisociality and general risk to offend. Understanding these broad patterns can help clinicians better match interventions to risk profiles, inform case formulation, and avoid overly homogenizing a heterogeneous population. For example, certain atypical sexual interests may be associated with higher rates of sexual offense specialization (e.g., Harris et al., 2009a).

Some authors have used trajectory methods to investigate whether the criminal careers of those who sexually offend differ from those who do not and whether those involved in sexual offenses are more likely to specialize in sexual offending (e.g., Babchishin et al., 2022; Francis et al., 2014; Lussier & Blokland, 2014; McCuish et al., 2016; McCuish & Lussier, 2017; Piquero et al., 2012; Reale et al., 2019). Prevailing fears surrounding adolescent-onset sexual offending have historically caused this group to be regarded as highly specialized and more likely to persistently sexually offend into adulthood (Lussier, 2017). These fears have, in part, led to the public endorsement of more stringent restrictions and harsher punishment practices when dealing with this group (Campregher & Jeglic, 2016; Chaffin, 2008). Contrary to these widespread beliefs, trajectory research has overwhelmingly disconfirmed these fears (e.g., Lussier & Blokland, 2014; McCuish et al., 2016; Piquero et al., 2012; Reale et al., 2019), at least when it comes to trajectories of officially detected offending.

### Adult-Onset Sexual Offending

The aggregate age-crime curve of sexual offending in Canada suggests that sexual offending peaks in both adolescence and middle adulthood (Canadian Centre for Justice Statistics, 1999; Hanson, 2002). This bimodal pattern of offending differs from the age-crime curve of general offending, which is characterized by a peak in offending in adolescence due to the emergence of a greater proportion of youth who participate in criminal behavior (e.g., Farrington, 1986). The second peak in the sexual offending age-crime curve may result from the emergence of a group of adults whose onset of sexual offending begins during this time.

Very little is known about offending trajectories amongst those who begin sexually offending in adulthood. Addressing this gap is essential to assess whether the later-onset offending reflects a transitory period or the emergence of a distinct pattern of chronic or escalating offending that is not captured by research focused on childhood and adolescent onset (e.g., Moffitt, 1993, 2018). For those who commit sexual offenses only as adults, do they engage in non-sexual offending as well, or do they show evidence of specialization in sexual offending? There has been evidence to suggest that their sexual offending may be motivated by a sexual interest in children (Francis et al., 2014; Lussier et al., 2010), a significant risk factor for persistent sexual offending (e.g., Mann et al., 2010).

Two studies have provided initial support for the existence of adult-onset sexual offending. Lussier et al. (2010) examined general offense trajectories between the ages of 12 and 35 in a sample of 250 men convicted of a sexual offense in Quebec between 1994 and 2000. Although they included individuals who began offending as adolescents, Lussier and colleagues found that only 12.8% of the sample were convicted of a crime before the age of 18. Further, two of the four identified trajectories were characterized by offending which began in adulthood. These two trajectories were the least criminally versatile in the sample and were most likely to specialize in sexual offending. One of the two offending trajectories began in early adulthood and escalated into their mid-30s, where their offending matched those who offended at the highest rate.

The second study was conducted by Francis et al. (2014), who identified two general offending trajectories which were characterized by adult-onset sexual offending. They performed their trajectory analyses on a sample of 489 men who were referred to a treatment center for sexual offending between 1959 and 1984. The two identified trajectories’ mean onset of offending occurred in their 20s and peaked in their 40s and 50s. One of the trajectories, labelled the late-onset escalators, was found most likely to sexually offend against children, and the majority of those in both trajectories had child victims.

### Present Study

Research on criminal careers of general offending suggests that later onset of offending is often associated with a short-lived pattern of low-rate offending (e.g., Piquero, 2008). However, studies examining official sexual offending data have noted that a subset of individuals whose offending starts in adulthood may escalate in their offending, a pattern that is more closely linked to persistent sexual offending against children (Francis et al., 2014; Lussier et al., 2010; McGee & Farrington, 2010). Consequently, it is important to examine whether the typically negative correlation between the age of onset and offending frequency might not be universally applicable to those involved in sexual offenses. This should include investigating whether individual-level factors can help explain the association between adult-onset sex offending and persistence. One potential explanation of escalation in adult-onset offending could be the association between adult-onset sex offending and the targeting of child victims, as having multiple child victims has been identified as an indicator of a sexual interest in children (Seto et al., 2017a) and a risk factor for persistent sexual offending in adulthood (Francis et al., 2014; Hanson, 2002).

Sexual interest in children, especially pedophilic or hebephilic interest, is believed to be stable in adulthood (Grundmann et al., 2016), and has been noted as a motivator and risk factor for sexual offending (Mann et al., 2010; Seto, 2019). Despite this, sexual interest in children does not directly translate to offending behavior, and its impact may be moderated by situational factors such as stress, opportunity, and substance use. Interestingly, the early life characteristics of those who begin offending in adulthood are similar to characteristics which have been identified in the early lives of those with a sexual interest in children, such as social difficulties, anxiety, and lower IQ (Beckley et al., 2016; Thornberry, 2010), which may serve as protective factors for early onset of offending. Notably, none of the previously described studies explicitly looked at whether those who begin sexually offending in adulthood are more likely to have a sexual interest in children.

The purpose of the present study was to analyze the general offense trajectories of men who had no history of offending in adolescence (sexual or non-sexual) and began sexually offending in adulthood. We aimed to examine whether there was heterogeneity in patterns of general offending among this subgroup. Given the limited research on this population, the first objective was to examine whether similar trajectory patterns would emerge as those observed in prior studies that have noted subgroups characterized by adult-onset sexual offending (Francis et al., 2014; Lussier et al., 2010; Piquero, 2008). Based on this literature, it was expected that four heterogeneous patterns would emerge: (1) The Rare/One-time trajectory, would consist of those who very rarely offend or are one-time offenders (commonly identified in trajectory research, e.g., Piquero, 2008); (2) the Emergent Adulthood trajectory, would be characterized by a higher frequency of offending at the outset (age 18) with desistance around age 25 at the end of emerging adulthood (Arnett, 2000; Sivertsson, 2018) representing a “delayed” adolescent-limited trajectory; (3) the Early Escalator trajectory, whose offending would begin sometime after emerging adulthood and then rapidly escalate through their life (Francis et al., 2014; Lussier et al., 2010); and, (4) the Late Escalator trajectory, whose offending would begin even later in adulthood (age 35+) before continuing to escalate through adulthood (Francis et al., 2014). Although the third and fourth expected trajectories represent a departure from the established age crime-curve (Farrington, 1986), they are supported by previously discussed research that sexual offending has a bimodal distribution, with peaks of offending occurring both in adolescence and middle adulthood (Canadian Centre for Justice Statistics, 1999; Hanson, 2002), and previously highlighted trajectory research (Francis et al., 2014; Lussier et al., 2010).

A secondary aim of the present study was to examine whether trajectory membership was associated with indicators of sexual interest in children, in line with findings by Francis et al., (2014), who identified that the late-onset trajectory had a higher likelihood of sexually offending against children. To reflect this, the second hypothesis was that membership to the Early and Late Escalator trajectories would be associated with the presence of child victims. The third hypothesis was that membership to the Early and Late Escalator trajectories would be associated with different indicators of sexual interest in children.

## Method

The present study received approval from relevant research ethics boards, and was preregistered on the Open Science Framework (Burgess et al., 2020). The project was originally registered as a MSc. thesis proposal and some of the original hypotheses were revised following committee feedback and further literature review. Changes from the pre-registered plan are noted below^1^. The authors take responsibility for the integrity of the data, the accuracy of the data analyses, and have made every effort to avoid inflating statistically significant results.

### Sample and Procedure

The present study used an archival dataset which consisted of 747 men who were charged with at least one sexual offense and were assessed at a large sexual behavior clinic in Toronto, Ontario between 1995 and 2006. Non-sexual and sexual offending data were collected using official offense records, and self-reported sexual offending was collected during a clinical interview. For study purposes, adult-onset sexual offending was defined as any individual who had no history of official or self-reported offending before the age of 18 and whose offending (inclusive of sexual offending) began in adulthood. In other words, the present study conservatively filtered out those with any history of offending prior to age 18, as sexual offenses can appear on criminal records as non-sexual offenses (Pedneault et al., 2015; Rice et al., 2006). For example, an attempted sexual assault by an adolescent might be pled down to nonsexual assault. In addition, some nonsexual offenses may be sexually motivated (e.g., burglary to obtain fetish items, trespassing as an attempted voyeuristic offense). Following the above inclusion criteria, 227 men were excluded due to offending prior to the age of 18, leaving 520 men included in the sample for analysis (See Table 1 for descriptives).

**Table 1.** Descriptive Statistics for Study Sample (*N* = 520)

Demographic variables	*M* (*SD*) or *n* (%)
Average age at assessment (*N* = 520)	42.7 (12.1)
Average age at first sexual offense (*N* = 520)	34.2 (11.5)
Any child victim (*n* = 519)	441 (84.8%)
One sexual offense victim (*N* = 520)	225 (49.0%)
Ethnicity (*n* = 517)	
White	394 (75.8%)
Black	37 (7.1%)
East Indian/Pakistani	25 (4.8%)
Aboriginal Canadian	9 (1.7%)
Filipino/Pacific Islander	12 (2.3%)
Asian	11 (2.1%)
Another ethnicity	29 (5.6%)
Education (*N* = 520)	
< Grade 12 education	228 (43.8%)
Grade 12 education	152 (29.2%)
> Grade 12 education	140 (26.3%)
Referral source (*n* = 508)	
Probation or Parole	259 (49.8%)
Institution	138 (26.5%)
Own lawyer	102 (19.6%)
Self referred	6 (1.2%)
Legal aid	3 (0.6%)

*Note.* Labels for ethnicity reflect how data was recorded in the dataset and may not align with current language regarding ethnicity.

Means and standard deviations are reported for continuous variables.

For categorical variables, frequencies and percentages are reported.

Average age at first known offense and the victim information based on information known at the time of assessment.

The dataset had been previously coded by one of the study authors and a trained research assistant as part of a larger study on recidivism. Interrater reliability was calculated on 10% of the cases using intraclass correlations (ICC), which were all above .90, except for violent recidivism (ICC = .75). The dataset has been used in previous studies addressing distinct research questions, and no study to date has examined criminal trajectories using this data (Faitakis et al., 2023; Monaghan et al., 2025; Moss et al., 2022; Stephens et al., 2017, 2018). A chronological count-based offense history was later created for each person, detailing the number of offenses which occurred during each age-year of their life, and the percentage of each year they spent outside of secure custody and in the community (time-at-risk, used to account for exposure time).

### Measures

#### Criminal Offending

The official offending records of all participants in the dataset were obtained from the Canadian Police Information Centre (CPIC) of the Royal Canadian Mounted Police. These records were received in September of 2013. Offenses on the CPIC were recorded as either charges, convictions, or both. Next, all offenses in the dataset were coded as contact sexual, non-contact sexual, serious violent, other violent, weapons, breach, property, or non-violent offense. All offenses captured in the dataset occurred between July 1953 and November 2012. The sentence length (in months) was also collected from official records to account for exposure time. At the time of the assessment, men were asked about the age of their first sexual offense, including undetected offending and offending that was not punished by the criminal legal system. Therefore, official information (i.e., CPICs) was supplemented with self-report data for the age of onset of sexual offending. Unfortunately, self-reported non-sexual offense history was not collected at the time of the assessment.

#### Criminal Career Parameters

Criminal career parameters were calculated using information obtained from CPIC records and information collected during the assessment to describe and compare the identified trajectories. The parameters included were as follows: age at which the individual was assessed; age at which the individual was charged for their first offense (inclusive of self-report and data from criminal records), regardless of offense type; age at which the individual committed their first sexual offense (inclusive of self-report and data from criminal records); total accumulated charges (lifetime total charges); total number of sexual offense charges; total contact sexual offenses; total non-contact sexual offenses; total number of non-sexual offense charges; total number of breach offenses; total number of incest related offenses; number of unique interactions (charges or convictions) with the criminal justice system; total time in months spent in custody over the study period; and total length of each individual’s criminal career in months (time between first and last offense).

#### Child Victim(s)

Victim information was collected based on a review of file information and self-reported victim information collected during the assessment. In cases of a discrepancy between these two sources of information, the source which resulted in the highest number of child victims was used. Child victims were operationalized as any victim below the age of 15 to best map onto prepubescent and pubescent children (Hames & Blanchard, 2012). Total number of child victims was capped at ten because some of the individuals had very high victim counts, resulting in skewed data.

#### Sexual Interest in Children

Three variables were used to assess the presence of sexual interest in children. The first indicator was based on phallometric testing, which measures sexual arousal via changes in penile blood volume during the presentation of sexual stimuli (Blanchard et al., 2001). The phallometric stimuli were visual depictions of males and females at various stages of sexual development (prepubescent, pubescent, and fully mature adults) and neutral stimuli (e.g., landscapes). Visual stimuli were accompanied by audio stimuli that consisted of short sexually explicit narratives related to the features of the person being visually depicted. Neutral audio narratives were also presented during neutral visual scenes (e.g., landscapes). The phallometric data were used to create a *pedohebephilia* (i.e., a term referring to sexual interest in prepubescent children or pubescent children) *index score* obtained by subtracting their greatest ipsatized response to adults from their greatest ipsatized response to either prepubescent or pubescent children. A positive index score indicated greater sexual interest in children relative to adults, whereas a negative index score indicated greater sexual interest in adults relative to children. A score of 0 on the index indicated equivalent arousal to children and adults. The pedohebephilia index was continuous. The sensitivity and specificity of the phallometric procedure in the present study are 75.3% and 90.7% for pedohebephilia (Cantor & McPhail, 2015).

The second indicator of sexual interest in children was based on a semi-structured sexual history interview conducted before phallometric testing and coded on a standardized form. During the interview, participants were asked to rank males and females by their preferred age range of attraction (0–5, 6–10, 11, 12–14, 15–16, 17+); each age range was ranked on a scale from one (strongest sexual interest) to five (least sexual interest). Individuals could assign the same value to multiple age and gender categories (i.e., tied scores were permitted). This ranking was reverse coded for ease of interpretation for the present study so that higher scores were reflective of a greater degree of sexual interest. Self-reported sexual interest in children is associated with increased numbers of child victims (Stephens et al., 2018; Woodworth et al., 2013) and is associated with sexual non-contact recidivism (Stephens et al., 2017).

The final indicator of sexual interest in children was a score on the Screening Scale for Pedophilic Interests Revised (SSPI-2; Seto et al., 2017a). The five items on the SSPI-2 are (1) boy victim, (2) more than one child victim, (3) victim aged 11 or younger, (4) unrelated child victim, and (5) child pornography. Each item is scored as present (1) or absent (0), and the total score ranges from zero to five. The association between the SSPI-2 and number of child victims was moderate (*r* = .68, *p* < .001, 95% CI [.63, .73]), indicating that they are related but distinct measures of sexual interest in children. The SSPI-2 is scored based on official file information and self-report victim information collected at the time of the assessment (i.e., it would not capture victim information after the assessment occurred). The SSPI-2 has good evidence for convergent and predictive validity (e.g., Faitakis et al., 2023; Seto et al., 2017a, 2017b; Stephens et al., 2019).

### Data Analysis

#### Trajectory Analysis

Group-based trajectory modelling is a non-parametric, nested modeling approach used to identify unknown trajectories in a longitudinal dataset (Nagin, 2005) and was carried out using the R package *crimCV* version 0.9.6 (Nielsen, 2018; Nielsen et al., 2014). The focus of the current study was on trajectories of general offending to allow for the investigation of how sexual offending fits within broader patterns of offending (e.g., sexual offense specialization or offending versatility). For the primary trajectory analyses, the chronological offense history and time-at-large data were capped at age 60 to balance the follow-up length with missing data (Reale et al., 2019; Sampson & Laub, 2003). Piquero’s (2008) review of research using trajectory methods found that the number of identified trajectories remains relatively stable over a sample size of 200. As the sample size does not fall below 200 until age 55 in this study, this level of attrition is unlikely to affect the reliability of the trajectories. modeled.

Trajectories were modeled beginning at age 18 for all participants, regardless of when their index sexual offense occurred. Person-periods between age 18 and the age of the index offense were retained and treated as having zero offenses, unless another official or self-reported offense occurred during that period. The index sexual offense and all subsequent offenses were included in the trajectory model. Missing data due to the end of follow up (e.g., aging out of the observation window) were assumed to be missing at random.

The trajectory analyses accounted for exposure time by measuring the proportion of each year of age that a person spent in the community as opposed to custody. Exposure values cannot equal zero and so instances in which persons spent an entire person-period observation in custody were adjusted to the equivalent of 10 days spent outside of custody. This inflation of exposure time is necessary to avoid model estimation issues in which an inordinate number of expected offenses is predicted (van der Geest et al., 2009).

The chronological count-based offense history served as the primary data for the trajectory analysis, and time-at-risk data was used to account for exposure time, which allows for the distinction between true desistance and nonoffending due to incarceration (Nagin, 2005). All models were conducted using 20 random sets of initialization values, as suggested by the author of the *crimCV* package (Nielsen, 2018). The optimal trajectory model was chosen based on a balance of Bayesian Information Criteria (BIC; Nagin, 2005) and the Cross-Validation Error (CVE), a metric recommended by the authors of the *crimCV* package which quantifies the error rate of the model when predicting alternating subsets of the data that was not used during the models training phase (Nielsen et al., 2014). The selected model was also required to display posterior probabilities of 0.70 or greater, odds of correct classification values of five or greater, and could not contain trajectories which contained less than 5% of the sample (Nagin, 2005; Reale et al., 2019).

#### Criminal Career Parameters Across Trajectories

A series of one-way ANOVAs were conducted to describe the identified trajectories by comparing them on commonly considered criminal career parameters. Welch’s ANOVAs were performed for each analysis due to violating the assumption of homogeneity, and Games-Howell post hoc tests followed up significant ANOVAs. Although some of these parameters (e.g., total criminal charges) are derived from the same age-year count data entered into the trajectory model, class membership is determined by the overall distribution of offences across the life span, not by any single summary statistic. Consequently, two trajectories could share identical total charges or onset age yet differ markedly in when offenses are concentrated.

#### Analysis of Child Victims and Indicators of Sexual Interest in Children

Multinomial logistic regression was used to predict trajectory membership based on the number of child victims and the three measures of sexual interest in children. The analyses for each variable of interest were conducted twice: first using the *Early Adulthood Escalator* trajectory as the reference and second using the *Late Escalator* trajectory as the reference. These variables are external to the age-year data entered into the trajectory analyses.

## Results

### Trajectory Analysis & Model Selection

Using the *crimCV* package in R, five models proposing one to five trajectories were estimated using a zero-inflated Poisson distribution to account for the high frequency of zero counts in the offending data (for a summary of model selection data, see Table 2). Although the CVE alone would suggest that a one-trajectory or two-trajectory model would best represent the data, the BIC continuously improved with added trajectories. Under ideal conditions, the BIC rewards parsimony in model prediction and would penalize for added complexity after a certain point (Nagin, 2005). Considered together, the CVE and BIC did not support a one or two-trajectory model to best fit the data. When considering the CVE of models with higher numbers of trajectories, the CVE improved from the three-trajectory model to the four-trajectory model but performed worse when moving from a four-trajectory to a five-trajectory model. This suggested that a four-trajectory model was most appropriate to represent the data. As further support for the retention of this model, previous research has indicated that three to five-trajectory models are often most appropriate for describing general offense trajectories (Piquero, 2008).

**Table 2.** BIC and Cross-Validation Error Values Used for Trajectory Model Selection

	BIC	CVE
# Of groups in model		
1	27921.60	0.71
2	24927.26	1.09
3	23517.52	1.61
4^a^	22584.82	1.53
5	21973.78	1.89
Four-group model equation		
Quadratic-linear	22671.79	4.54
Quadratic-quadratic	22667.07	2.11
Quadratic-cubic	22750.62	1.96
Cubic-linear	22560.91	1.76
Cubic-quadratic^b^	22557.86	1.59
Cubic-cubic	22584.82	1.53

*Note.* BIC = Bayesian Information Criteria; CVE = Cross-validation error.

^a^Number of groups in the final model.

^b^Functional form of final model equation.

Following the choice of a four-trajectory model, four additional models were tested to determine the optimal functional form of the likelihood equation (i.e. quadratic-quadratic, quadratic-cubic, cubic-linear, and cubic-quadratic; Nielsen et al., 2014). To illustrate, “cubic-quadratic” refers to the use of a cubic polynomial function to model the offense mean trajectories (log-linear component) and a quadratic polynomial function to model the zero-inflation probabilities (logistic component). Unlike ProcTraj in Mplus, *crimCV* cannot specify individual orders (cubic, quadratic, or linear) of individual trajectories in the model. Based on the optimal BIC value, the cubic-quadratic equation was chosen as the best four-trajectory model. For the selected model, all odds of correct classification values were greater than five, all average posterior probabilities were greater than 0.70, and no trajectory contained less than 5% of the sample (see Table 3).

**Table 3.** Fit Statistics for Four-Group Trajectory Model (N = 520)

	Offending trajectories			
	G1: Early adulthood escalators	G2: Late escalators	G3: Low-level intermittent	G4: Low-level chronic
*n (%)*	70 (13.46%)	83 (15.96%)	145 (27.89%)	222 (42.69%)
Median PP	0.97	0.92	0.92	0.99
Probability range	0.39–1.00	0.49–1.00	0.47–1.00	0.37–1.00
Mean probability: G1	0.89	0.03	0.01	0.07
Mean probability: G2	0.02	0.85	0.01	0.13
Mean probability: G3	0.03	0.05	0.86	0.07
Mean probability: G4	0.02	0.06	0.02	0.91
OCC	7.98	5.60	6.10	10.07

*Note.* G1 = Group 1; G2 = Group 2; G3 = Group 3; G4 = Group 4; Median PP = median posterior probability of those assigned to group; OCC = odds of correct classification into group.

**Figure 1.**
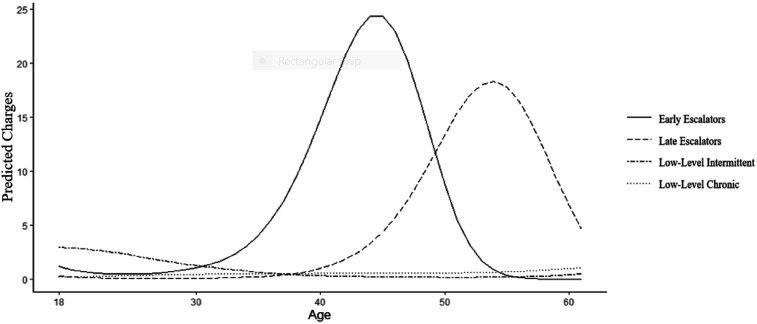
General offending trajectories of adult-onset sexual offenders. The figure displays the predicted number of charges per year by trajectory group membership

The identified trajectories are plotted in Figure 1 and generally resemble the expected patterns outlined in the present study section. The first trajectory identified, labelled the *Early Adulthood Escalator* trajectory (13.5% of the sample), was characterized by a low rate of offending throughout their 20s followed by a rapid escalation of offending in the early 30s, which peaked in the mid-40s and began to deescalate over the remainder of the follow-up period. The second trajectory, labelled the *Late Escalator* trajectory (16.0% of the sample), demonstrated a similar trajectory shape to the *Early Adulthood Escalator* trajectory; however, they had a later offense onset and peak, which occurred in the mid-40s and mid-60s, respectively. Therefore, the pattern between the *Early* and *Late Adulthood Escalator* trajectories differed in the timing of their offending. The offending peak was not as high as in the *Early Adulthood Escalator* trajectory. The third trajectory identified, labelled the *Low-Level Intermittent* trajectory (27.9% of the sample), was characterized by an uptick of offending at the outset of adulthood, which slowly desisted until becoming a pattern of sporadic offending throughout adulthood. The fourth trajectory, labelled the *Low-Level Chronic* trajectory (42.7% of the sample), was characterized by an intermittent pattern of offending spread at a consistently low rate across the study period.

### Criminal Career Parameters Across Trajectories

A series of one-way analysis of variance (ANOVA) tests were conducted to describe and contrast the four identified trajectories on criminal career parameters (See Table 4). Because many of the parameters were positively skewed, we also examined medians and interquartile ranges (see Supplemental Table 1). While these analyses were not tied to specific hypotheses, they were conducted to provide further context to the identified trajectories and characterize the trajectories beyond visual inspection. Prior to the main analyses, we examined whether follow-up time differed based on trajectory membership, to ensure that individuals with shorter follow-up time would not be predisposed to being placed in trajectories characterized by short-term offense patterns. Follow-up time differed significantly (*F* (3) = 36.89, *p* < .001), as members of the *Early Adulthood Escalator* trajectory (*M* = 44.44, *SD* = 10.23) had significantly shorter follow-up than all other trajectories. Although the *Low-Level Chronic* trajectory (*M* = 48.74, *SD* = 8.87) did not differ from the *Late Adulthood Escalator* trajectory (*M* = 50.65, *SD* = 6.38), they both displayed significantly shorter follow-up time than the *Low-Level Intermittent* trajectory (*M* = 55.82, *SD* = 6.65). The remaining parameter comparisons were conducted using the length of follow-up as a covariate, considering the potential impact of follow-up time on the remaining criminal career parameters (Table 4).

**Table 4.** Criminal Career Parameters of Four Offending Trajectories of Adult-Onset Sexual Offenders

Criminal career parameters	Early adulthood escalators (*n* = 70)	Late escalators (*n* = 83)	Low-level intermittent (*n* = 122)	Low-level chronic (*n* = 222)	Omnibus welch test, ω^2^
Age at assessment	35.3 (10.8)	41.8 (7.8)^a^	51.7 (11.9)^a,b^	39.5 (10.6)^a,c^	*F* (3, 211.5) = 45.3^***^, ω^2^ = .20
Age of first official offense	29.9 (11.0)	33.3 (10.3)	45.6 (9.4)^a,b^	28.6 (8.9)^b,c^	*F* (3, 189.6) = 92.7^***^, ω^2^ = .35
Age of first sexual offense	26.3 (7.3)	32.9 (7.2)^a^	39.3 (11.0)^a,b^	31.0 (8.7)^a,c^	*F* (3, 210.7) = 34.9^***^, ω^2^ = .16
Lifetime total charges	13.8 (15.7)	10.5 (14.6)	6.5 (8.4)^a^	11.6 (9.7)^c^	*F* (3, 184.8) = 11.4^***^, ω^2^ = .06
Total sexual offenses	7.1 (6.7)	6.7 (7.6)	4.6 (6.3)^a^	4.5 (4.1)^a^	*F* (3, 178.7) = 4.9^*^, ω^2^ = .02
Contact sexual offense	5.8 (5.6)	5.8 (7.2)	4.3 (6.3)	4.1 (3.8)	*F* (3, 180.2) = 3.0^*^, ω^2^ = .01
Non-contact sexual	1.2 (3.3)	0.9 (3.1)	0.3 (0.9)	0.4 (1.2)	*F* (3, 174.7) = 2.8^*^, ω^2^ = .004
Total incest victims	0.4 (0.8)	0.6 (1.0)	0.4 (0.6)	0.5 (0.8)	*F* (3, 198.1) = 1.9, ω^2^ = .005
Total non-sexual offenses	6.7 (13.9)	3.8 (9.6)	1.9 (5.7)^a^	7.1 (8.7)^b,c^	*F* (3, 186.9) = 16.9^***^, ω^2^ = .08
Total breach offenses	1.9 (5.1)	1.2 (5.1)	0.3 (0.8)	1.4 (2.3)^c^	*F* (3, 169.3) = 17.6^***^, ω^2^ = .09
Unique court contacts	3.4 (4.9)	2.6 (2.8)	1.7 (1.3)^a,b^	5.0 (4.1)^b,c^	*F* (3, 180.9) = 42.8^***^, ω^2^ = .19
Total time in custody	41.5 (73.3)	18.6 (24.3)	11.4 (30.2)^a^	13.6 (17.7)^a^	*F* (3, 175.1) = 4.6^*^, ω^2^ = .02
Length of criminal career	5.5 (7.9)	8.3 (10.3)	3.2 (6.0)^b^	13.1 (10.4)^a,b,c^	*F* (3, 201.6) = 45.3^***^, ω^2^ = .20

*Note.* Means and standard deviations are reported. Total time in custody and length of criminal career reported in months. Homogeneity of variance is violated, and Welch’s test is used in all analyses.

ω^2^ Effect size = Omega squared.

*p < .05, ***p < .001.

^a^Indicates significantly different from Early Adulthood Escalator.

^b^Indicates significantly different from Late Escalator.

^c^Indicates significantly different from Low-Level Intermittent.

In brief, the general trends identified were as follows: The *Early Adulthood Escalator* trajectory had the youngest age of sexual offense onset, accrued the most sexual offenses (including when considering contact and non-contact separately) and overall charges, spent the longest amount of time in secure custody, and had the greatest number of breach charges. The *Low-Level Intermittent* trajectory was the oldest at assessment, the oldest for official general and self-reported sexual offense onset, had the fewest sexual offense charges, unique court contacts, and breach offenses; spent the least time in secure custody and had the shortest criminal careers. The *Low-Level Chronic* trajectory had the youngest age at official offending onset, the longest criminal careers, the most unique court contacts, committed the most non-sexual offenses, and the least sexual offenses. The presence of incest victims was not associated with any specific trajectory. When examining median and interquartile ranges, the overall pattern of group differences was largely unchanged: The *Early Adulthood Escalator* and *Late Escalator* trajectories showed earlier onset and higher offending frequency than *Low-Level Intermittent* trajectory, whereas the *Low-Level Chronic* trajectory demonstrated the longest criminal careers.

### Analysis of Child Victims and Indicators of Sexual Interest in Children

A series of multinomial logistic regressions were conducted to test the remaining hypotheses regarding the presence of child victims and indicators of sexual interest in children (see Tables 5 and 6 for descriptives and regression results). The hypothesis that the presence of child victims would be associated with membership to either the *Early Adulthood Escalator* or the *Late Adulthood Escalator* trajectories compared with the other trajectories was supported (*χ*^*2*^ (3) = 17.60, *R*^*2*^_*McF*_ = 0.013, *p* < .001). Specifically it was found that with increasing numbers of child victims, men were more likely to belong to the *Early Adulthood Escalator* trajectory over that of the *Low-Level Intermittent* (*OR* = 1.17, *95% CI* = 1.02, 1.34, *p* = .028) and *Low-Level Chronic* (*OR* = 1.31, *95% CI* = 1.13, 1.51, *p* < .001) trajectories. Further, with increasing numbers of child victims, men were more likely to belong to the *Late Adulthood Escalator* trajectory over the *Low-Level Chronic* trajectory (*OR* = 1.25, *95% CI* = 1.09, 1.44, *p* = .002).

**Table 5.** Descriptives for Indicators of Sexual Interest in Children Across Trajectory Groups

Child victims/Indicators of sexual interest in children	Early adulthood escalators (*n* = 70)	Late escalators (*n* = 83)	Low-level intermittent (*n* = 122)	Low-level chronic (*n* = 222)
Any child victim *n* (%)	62 (88.6)	73 (87.9)	128 (88.3)	175 (78.8)
Total child victims	2.4 (2.5)	2.1 (2.1)	1.7 (1.7)	1.4 (1.5)
Pedohebephilia index score	0.19 (0.87)	0.05 (0.70)	0.04 (0.79)	−0.12 (0.79)
Self-reported sexual interest	4.1 (1.4)	4.4 (1.1)	4.4 (1.2)	4.6 (0.9)
SSPI-2 scores	2.3 (1.5)	2.0 (1.4)	1.9 (1.3)	1.6 (1.3)

*Note.* Mean and standard deviation reported, except % for presence of child victims and interquartile ranges are reported. Self reported sexual interest rated on scale of 1 (highest sexual interest) to 5 (least sexual interest).

SSPI-2 = Screening Scale for Pedophilic Interests – Revised.

**Table 6.** Relative Risk Ratios Predicting Trajectory Membership Using Indicators of Sexual Interest in Children and Presence of Child Victims (*N* = 520)

Reference group	Child victims/Indicators of sexual interest in children			
Early adulthood escalator	**Child victim**	***χ*** ^***2***^ ** (3) ** **= 17.60, ** ***R*** ^***2***^_***McF***_ **= 0.013, ** ***R*** ^***2***^_***N***_ **= 0.018, ** ***p*** **< .001**		
		*b (SE)*	*RRR*	*95% CI*
	Late escalator	−0.04 (0.07)	0.96	0.83, 1.10
	Low-level intermittent	−0.15 (0.07)	0.86*	0.74, 0.98
	Low-level chronic	−0.27 (0.07)	0.77***	0.67, 0.88
	**Pedohebephilia index score**	***χ*** ^***2***^ ** (3) ** **= 9.85, ** ***R*** ^***2***^_***McF***_ **= 0.01, ** ***R*** ^***2***^_***N***_ **= 0.02, ** ***p*** **= .020**		
		*b (SE)*	*RRR*	*95% CI*
	Late escalator	−0.23 (0.21)	0.79	0.53, 1.20
	Low-level intermittent	−0.23 (0.19)	0.79	0.55, 1.15
	Low-level chronic	−0.50 (0.18)	0.61*	0.43, 0.86
	**Self-** **reported** ** sexual interest**	***χ*** ^***2***^ ** (3) ** **= 10.80, ** ***R*** ^***2***^_***McF***_ **= 0.01, ** ***R*** ^***2***^_***N***_ **= 0.01 ** ***p*** **= .013**		
		*b (SE)*	*RRR*	*95% CI*
	Late escalator	−0.18 (0.13)	0.84	0.65, 1.08
	Low-level intermittent	−0.15 (0.11)	0.86	0.69, 1.07
	Low-level chronic	−0.36 (0.11)	0.70***	0.56, 0.87
	**SSPI-** **2 scores**	***χ*** ^***2***^ ** (3) ** **= 14.36, ** ***R*** ^***2***^_***McF***_ **= 0.01, ** ***R*** ^***2***^_***N***_ **= 0.03, ** ***p*** **= .002**		
		*b (SE)*	*RRR*	*95% CI*
	Late escalator	−0.15 (0.12)	0.86	0.69, 1.09
	Low-level intermittent	−0.20 (0.11)	0.82	0.66, 1.00
	Low-level chronic	−0.35 (0.10)	0.70***	0.58, 0.86
Late escalators	**Child victim**	***χ*** ^***2***^ ** (3) ** **= 17.60, ** ***R*** ^***2***^_***McF***_ **= 0.013, ** ***R*** ^***2***^_***N***_ **= 0.018, ** ***p*** **< .001**		
		*b (SE)*	*RRR*	*95% CI*
	Early adulthood escalator	0.05 (0.07)	1.05	0.91, 1.20
	Low-level intermittent	−0.11 (0.07)	0.90	0.78, 1.03
	Low-level chronic	−0.22 (0.07)	0.80*	0.70, 0.92
	**Pedohebephilia index scores**	***χ*** ^***2***^ ** (3) ** **= 9.85, ** ***R*** ^***2***^_***McF***_ **= 0.01, ** ***R*** ^***2***^_***N***_ **= 0.02, ** ***p*** **= .020**		
		*b (SE)*	*RRR*	*95% CI*
	Early adulthood escalator	0.23 (0.21)	1.26	0.84, 1.90
	Low-level intermittent	−0.001 (0.18)	1.00	0.71, 1.41
	Low-level chronic	−0.27 (0.17)	0.76	0.55, 1.06
	**Self-** **reported** ** sexual interest**	***χ*** ^***2***^ ** (3) ** **= 10.80, ** ***R*** ^***2***^_***McF***_ **= 0.01, ** ***R*** ^***2***^_***N***_ **= 0.01 ** ***p*** **= .013**		
		*b (SE)*	*RRR*	*95% CI*
	Early adulthood escalator	0.18 (0.13)	1.20	0.93, 1.54
	Low-level intermittent	0.03 (0.12)	1.03	0.82, 1.30
	Low-level chronic	−0.18 (0.12)	0.84	0.66, 1.06
	**SSPI-** **2 scores**	***χ*** ^***2***^ ** (3) ** **= 14.36, ** ***R*** ^***2***^_***McF***_ **= 0.01, ** ***R*** ^***2***^_***N***_ **= 0.03, ** ***p*** **= .002**		
		*b (SE)*	*RRR*	*95% CI*
	Early adulthood escalator	0.15 (0.12)	1.16	0.92, 1.46
	Low-level intermittent	−0.06 (0.10)	0.95	0.78, 1.15
	Low-level chronic	−0.21 (0.10)	0.81*	0.68, 0.98

*Note.* RRR = Relative risk ratio. Reporting dichotomized self-reported sexual interest.

SSPI-2 = Screening Scale for Pedophilic Interests – Revised.

*p < .05, ***p < .001.

For the third hypothesis, it was expected that all three indicators of sexual interest in children would be associated with membership to both the *Early Adulthood Escalator* and the *Late Adulthood Escalator* trajectories compared with the other trajectories. Indeed, all three indicators of sexual interest in children were found to be associated with membership to the *Early Adulthood Escalators* over the *Low-Level Chronic* trajectory, and higher scores on the SSPI-2 were associated with membership to the *Late Adulthood Escalator* trajectory over the *Low-Level Chronic* trajectory.

## Discussion

In the present sample, adult-onset sexual offending is best represented by a four-trajectory model, which captures the criminal careers of a sample of men whose offending began in adulthood and included at least one sexual offense. These trajectories were closely aligned with previously identified patterns of adult-onset sexual offending (e.g., Francis et al., 2014; Lussier et al., 2010). The findings underscore that sexual offending is a heterogeneous and multifactorial phenomenon with distinct patterns reflecting differences in offending, criminal career parameters, and risk relevant factors such as sexual interest in children.

Both the *Early* and *Late Adulthood Escalator* trajectories are similar to patterns of offending first identified in previous research (Francis et al., 2014; Lussier et al., 2010). In both previous studies, the authors identified at least one trajectory whose offending began in early adulthood and appeared to escalate over time. In the present study just under 30% of individuals were assigned to these trajectories. Like past research, these patterns were not limited to just sexual offending (Lussier et al., 2010). Although Lussier et al. (2010) found only one escalating trajectory, the fact that both the present study and Francis et al. (2014) identified two escalating patterns of offending highlights the importance of long-term follow-up periods for understanding the complete picture of offending across the lifespan. Shorter follow-up periods may fail to identify crucial turning points that occur later in the lifespan, such as those exhibited by the *Early* and *Late Adulthood Escalator* trajectories identified in the present study.

Like the escalating trajectories identified by Francis et al. (2014), the *Early* and *Late Adulthood Escalator* trajectories were found to exhibit higher rates of sexual offending and had higher odds of sexually offending against a child than other identified trajectories. Further, indicators of a sexual interest in children were associated with the *Early Escalator* trajectory, which was characterized by both an earlier onset of offending and the greatest number of sexual offense charges (contact and non-contact). Given the association between indicators of sexual interest in children and the two *Escalator* trajectories, it is noteworthy that their sexual offending appears to begin in adulthood rather than earlier in development.

Although paraphilias, inclusive of sexual interest in children, are powerful motivators of sexual offending (Mann et al., 2010; Seto, 2019) other factors such as substance use, impulsivity, coercive sexual interests (Seto, 2018, 2019), or limited access to consensual sexual outlets (Finkelhor et al., 2016) can also contribute to offending onset. Adult-onset sexual offending may also reflect increased opportunity, as unsupervised access to children becomes more likely with age, and early protective factors such as school involvement, overbearing home lives, social isolation, and delayed pubertal onset (Blanchard & Dickey, 1998; Gibson & Krohn, 2013) recede. Another explanation for this finding could be detection avoidance (Nicol et al., 2022). For example, Lussier and Mathesius (2012) reported an average of seven years between a sexual offense and official adjudication for that offense; however, studies of general offending suggest that true adult-onset of offending is not merely an artifact of administrative data (e.g., Matsuda et al., 2022). If adult-onset sexual offending is not a mirage, understanding why it emerges when most individuals are desisting from crime (Sampson & Laub, 2003) remains important.

The other two identified trajectories, the *Low-Level Intermittent* and *Low-Level Chronic* trajectories, were less likely to have child victims and were not associated with sexual interest in children; just over 70% of the sample were assigned to these two trajectories. The *Low-Level Intermittent* trajectory had the oldest average onset of both their sexual and official offending, the fewest unique court contacts and sexual offenses, and the shortest overall criminal careers. These findings place the *Low-Level Intermittent* trajectory in line with low-level to one-time offending trajectories, which are commonly found in trajectory research (Francis et al., 2014; Lussier et al., 2010; McCuish et al., 2016; Reale et al., 2019). Given this trajectory’s low number of unique court contacts, it is unlikely that they would be considered to specialise in any one type of offending, including sexual offending.

The *Low-Level Chronic* trajectory is noteworthy as it constitutes the largest portion of the study sample (43%). This trajectory displayed a persistent low-moderate level of offending throughout adulthood, experienced the longest criminal career, the youngest age of official offending onset, and committed the most non-sexual offenses of all the identified trajectories. These findings suggest that members of the *Low-Level Chronic* trajectory may exhibit a general pattern of antisociality and are less likely to specialize in sexual offending, in contrast to the *Early Adulthood Escalator* trajectory.

### Theoretical and Applied Implications

The trajectories identified in this study underscore the heterogeneity of adult-onset sexual offending and offer some potential insights for assessment and case formulation. Notably, these findings highlight that a “one-size-fits-all” case formulation approach for those who sexually offend against children is likely not advisable (Gillespie & Elliott, 2023). The presence of two *Escalator* trajectories, both associated with increased likelihood of having child victims, indicators of sexual interest in children, and more extensive sexual offending (in terms of frequency of sexual charges), may be at higher risk for sexual offending against children. One factor that can explain patterns of specialization in sexual offending (as reflected by the higher frequency of sexual charges) is the presence of atypical sexual interests, which are important motivators of sexual offenses (Harris et al., 2009b). Nonsexual factors (e.g., substance use, impulsivity) can impact sexual offending as well (Seto, 2019). The implications of these findings in the context of that wider literature suggest that some individuals who begin offending in adulthood may present as higher risk for persistent sexual offending and necessitate more specialized risk management considerations.

These findings also have implications for risk conceptualization. If the trajectory patterns identified here are reproduced using more confirmatory research methods, they may help inform expectations of how an individual’s offending could evolve over time, much like how broader models of adolescent criminal offending can shape expectations of chronicity of offending (e.g., Moffitt, 1993). The fact that the two *Escalator* trajectories had higher odds of offending against children could suggest that certain configurations of risk factors may be associated with more persistent or escalating patterns of offending. At the same time, the presence of these factors across multiple trajectories highlights that no single, or even cluster, of risk factors is sufficient to determine an individual’s criminal pathway. Relying on a baseline measure of a risk factor, while useful, neglects to consider within-individual change over time, particularly for dynamic risk factors such as substance use, or how other internal or external influences (e.g., involvement in treatment, emerging protective factors) might alter the relevance of that risk factor. Together, these findings underscore the importance of a nuanced dynamic approach to risk conceptualization that considers not only what factors are present, but how they may evolve and interact to shape offending over time.

In contrast, the two *Low-Level* trajectories represented 70% of the sample and were not associated with indicators of sexual interest in children. In particular, the *Low-Level Chronic* trajectory had the longest criminal career and greatest number of court contacts suggesting that a notable number of individuals who are adult-sexual offenders are more engrained in their offending and appeared to follow a generalization pathway in which their offending behavior is more versatile and may be more reflective of general antisociality (e.g., Harris et al., 2009a, 2009b; Lussier, 2005).

Lastly, it is notable that 85% of the sample had offended against a child victim, which was a roughly consistent proportion across trajectories, but was highest when sexual interest in children was present. This reinforces the idea that many individuals who offend against children may not be driven by pedohebephilic sexual interests, but rather may be doing so as part of a general disposition for offending and require interventions that more broadly target antisociality (Seto, 2018).

### Limitations

The present study primarily relied on official criminal records for the trajectory analyses, which poses certain limitations. First, relying on official records underestimates the actual rate of offending in a population (Eggleston & Laub, 2002; Farrington et al., 2014), which is problematic for trajectory analyses, particularly for the accurate identification of adult-onset sexual offending for inclusionary purposes. The issue of underreporting is especially relevant in the study of sexual offending, where behaviors often go undetected for years before being reported (Seto & Eke, 2015). As a result, when sexual offenses are finally reported, they may appear as a cluster of charges covering an extended period, including historical offenses, which may not accurately reflect an individual’s true pattern of offending. While the present study includes self-reported age of onset for sexual offending, some individuals may be unwilling to disclose past sexual offending as doing so could expose them to legal consequences or trigger mandatory reporting.

Additionally, official records may be systematically hidden through processes like record suspensions, which in Canada can be granted after five and ten years for summary and indictable offenses, with separate rules for youth records (Criminal Records Act, 1985; Youth Criminal Justice Act, 2002) It should be noted that these specific concerns are not unique to this project and underscore the need to address systemic issues in data collection related to offending behavior, particularly sexual offending. Researchers should advocate for more nuanced approaches to the recording of sexual offenses to more clearly differentiate between the age of official onset/detection and the actual onset of offending behavior.

Another limitation concerns the composition of the sample, as those who committed a sexual offense against a child comprised (∼85%) of the sample. Therefore, the findings taken from this study may not be generalizable to those who commit sexual offenses against adults. Similarly, many of the sample were referred and took part in treatment, which may have altered their overall offending trajectories. A related limitation is that participants are drawn from multiple birth cohorts, with offenses spanning the mid-1950s through the early 2010s. Such temporal breadth likely captures cohort and period effects such as shifts in social attitudes towards crime, which can impact birth cohorts growing up in certain social contexts (Neil et al., 2021). For example, older cohorts show higher sexual recidivism than more recent ones (Lussier et al., 2023). Additionally, most of our sample was assessed between 2000 and 2006, before broadband internet, social media, and smartphones became ubiquitous, thereby limiting opportunities for technology-mediated offending (Beech et al., 2008; Lussier et al., 2023). More recent trends show that rates of online sexual offending have increased substantially (Seto, 2025), which may not be captured by the present data.

Lastly, GBTM assumes homogeneity within groups and does not model within-group variance, which may oversimplify individual differences in offending behavior (Mésidor et al., 2022), or risk overfitting the model. This can be problematic in real-world samples where behavioral trajectories likely vary substantially. Although multiple model fit indices were used to help mitigate these concerns, and the model partially replicated trajectories identified in previous research while differing in meaningful ways on commonly considered criminal career parameters, it remains possible that some of the identified trajectories are oversimplified. It is also possible that alternative modeling approaches (e.g., latent class, cluster modeling, sequence analysis, etc.) could identify different patterns of offending behavior. Additionally, GBTM approximates rather than reveals a “true” number of trajectory groups, meaning that identified subgroups are best interpreted as practical approximations of underlying patterns, not fixed categories (Nagin et al., 2024).

More broadly, the present analyses represent an early step toward understanding patterns of offending among a population that has received limited empirical attention. As such, the modeled trajectories are not intended to make direct causal, or etiological claims. That said, despite limitations of our methods, this study provides a useful heuristic framework for understanding these patterns.

### Future Research Directions

The present study represents an early step towards understanding adult-onset sexual offending patterns. An important direction for future research is to replicate and extend the present findings using a set of true lifetime or prospective longitudinal data. Doing so is crucial for determining the generalizability of the *Early* and *Late Escalator* trajectories identified here. These trajectories are not only associated with an escalation of offending over time but also appear to be more likely to sexually offend against children, show a higher likelihood of sexual interest in children, and are more likely to breach conditions in the community. These features suggest a higher risk profile overall and underscore the need for further investigation, especially related to early detection and intervention. All trajectories, but especially the *Late Escalator* trajectory spent time incarcerated. Criminal legal system intervention may have altered for better or worse, criminogenic risk factors including sexual interest in children. The current study did not capture potential changes in such risk factors that could have implications for a person’s offending trajectory. Future research would particularly benefit clinical practices by examining whether stability or change in these risk factors alters the course of a person’s offending trajectory.

Given the resource and time-intensive nature of true prospective longitudinal studies alongside the challenge of identifying those whose sexual offending begins in adulthood prior to offending onset, identification of these trajectories in additional datasets is a crucial next step. This step is necessary to determine whether these trajectories are statistical artifacts, the result of delays between offending and detection, or a true subset of those who sexually offend. As online access and technology-facilitated offending have continued to evolve (see Seto, 2025 for a comprehensive overview), future research emphasizing more recent cohorts and offending behaviors may provide insight into how these societal shifts affect offending trajectories in this population.

## Supplemental Material

Supplemental Material - Offending Trajectories of Men With Adult-Onset Sexual Offending Histories Supplemental Material for Offending Trajectories of Men With Adult-Onset Sexual Offending Histories by Brandon A. Burgess, Skye Stephens, Evan McCuish, Michael C. Seto in Sexual Abuse

## Note

1. The general shape of expected trajectories were unchanged, but the names were changed to better reflect the described patterns. The second pre-registered hypothesis originally looked at associations between trajectories and various victim types (e.g., incest and peer victims), however this was modified to solely analyze the number of child victims, thereby focusing on patterns reflective of the previously identified late-onset trajectories (Francis et al., 2014; Lussier et al., 2010). Additionally, the number of child victims was analyzed continuously and capped at ten, rather than dichotomously to avoid losing variance in analysis. The third hypothesis remained unchanged.
