# Supplementary material for: Offending Trajectories of Men With Adult-Onset Sexual Offending Histories
Source: Sex Abuse. 2026 Feb 28;38(4):474–500. doi: 10.1177/10790632261429126 (PMC13083822; doi:10.1177/10790632261429126)
Supplement: Supplemental Material - Offending Trajectories of Men With Adult-Onset Sexual Offending Histories [file sj-pdf-1-sax-10.1177_10790632261429126.pdf]

**Supplemental Table 1.**

*Criminal Career Parameters of Four Offending Trajectories of Adult-Onset Sexual Offenders*

| Criminal Career Parameters | Early Adulthood Escalators (*n* = 70) | Late Escalators (*n* = 83) | Low-Level Intermittent (*n* = 122) | Low-Level Chronic (*n* = 222) |
|---|---|---|---|---|
| Age at assessment | 34.0(26.0 – 43.3) | 40.0(36.0 – 47.0)[a] | 48.0(43.0 – 56.0)[a,b] | 39.0(31.3 – 47.0)[a,c] |
| Age of first official offense | 26.0(21.0 – 40.3) | 34(19.0 – 38.0) | 45.5(41.3 – 52.8)[a,b] | 26.5(21.0 – 35.0)[b,c] |
| Age of first sexual offense | 25.0(20.0 – 32.0) | 33.0(29.0 – 37.0)[a] | 39.0(31.3 – 45.8)[a,b] | 30.0(24.0 – 38.0)[a,c] |
| Lifetime total charges | 7.5(5.0 – 16.0) | 7.0(3.0 – 11.0) | 4.0(2.0 – 7.0)[a] | 8.0(4.0 – 16.0)[c] |
| Total sexual offenses | 5.5(3.0 – 8.0) | 4.0(2.0 – 8.0) | 3.0(2.0 – 5.0)[a] | 3.0(2.0 – 6.0)[a] |
| Contact sexual offense | 4.5(3.0 – 6.3) | 4.0(2.0 – 6.0) | 3.0(2.0 – 4.0) | 3.0(2.0 – 5.0) |
| Non-contact sexual | 0(0.0 – 1.0) | 0.0(0.0 – 1.0) | 0.0(0.0 – 0.0) | 0.0(0.0 – 0.0) |
| Total incest victims | 0.0(0.0 – 1.0) | 0.0(0.0 – 1.0) | 0.0(0.0 – 1.0) | 0.0(0.0 – 1.0) |
| Total non-sexual offenses | 1.0(0.0 – 8.5) | 1.0(0.0 – 3.0) | 0.0(0.0 – 1.0)[a] | 4.0(1.0 – 10.8)[b,c] |
| Total breach offenses | 0.0(0.0 – 1.0) | 0.0(0.0 – 0.0) | 0.0(0.0 – 0.0) | 0.0(0.0 – 2.0)[c] |
| Unique court contacts | 2.0(2.0 – 4.0) | 2.0(1.0 – 3.0) | 1.0(1.0 – 2.0)[a,b] | 3.5(2.0 – 7.0)[b,c] |
| Total time in custody | 20.0(12.0 – 38.6) | 14.0(0.5 – 24.0) | 2.0(0.0 – 13.9)[a] | 6.8(0.0 – 20.9)[a] |
| Length of criminal career | 2.5(0.0 – 9.0) | 3.0(0.0 – 17.0) | 0.0(0.0 – 3.8)[b] | 12.0(4.0 – 20.0)[a,b,c] |

*Note:* Median and interquartile ranges are reported. Total time in custody and length of criminal career reported in months. Significant differences from Table 4. maintained for sake of comparison.

[a] Indicates significantly different from Early Adulthood Escalator

[b] Indicates significantly different from Late Escalator

[c] Indicates significantly different from Low-Level Intermittent

$^{*}$ $p < .05$, $^{***}$ $p < .001$
